# Abrocitinib for treatment of solid facial edema

**DOI:** 10.1016/j.jdcr.2025.02.001

**Published:** 2025-03-01

**Authors:** Axel De Greef, Marie Baeck

**Affiliations:** a Department of Dermatology, Cliniques universitaires Saint-Luc, Université catholique de Louvain (UCLouvain), Brussels, Belgium; b Institute of Experimental and Clinical Research (IREC), Pneumology, ENT and Dermatology Pole (LUNS), Université catholique de Louvain (UCLouvain), Brussels, Belgium

**Keywords:** abrocitinib, JAK inhibitor, Morbihan syndrome, solid facial edema

## Introduction

Solid facial edema (SFE), or Morbihan syndrome, is a rare, disfiguring, and difficult-to-treat condition characterized by the gradual onset of erythema and solid edema affecting the upper portion of the face.^1^^,^^2^ It is considered a complication or late-stage manifestation of rosacea or acne. The involvement of perilymphatic granulomas has been suspected in the pathogenesis of SFE,^3^^,^^4^ and Janus kinase (JAK) inhibitors have recently shown efficacy in treating cutaneous granulomatous diseases.^5^ We report the cases of 3 patients with SFE who demonstrated substantial improvement after treatment with abrocitinib (JAK 1 inhibitor).

## Case report

### Case 1

**Fig 1:**
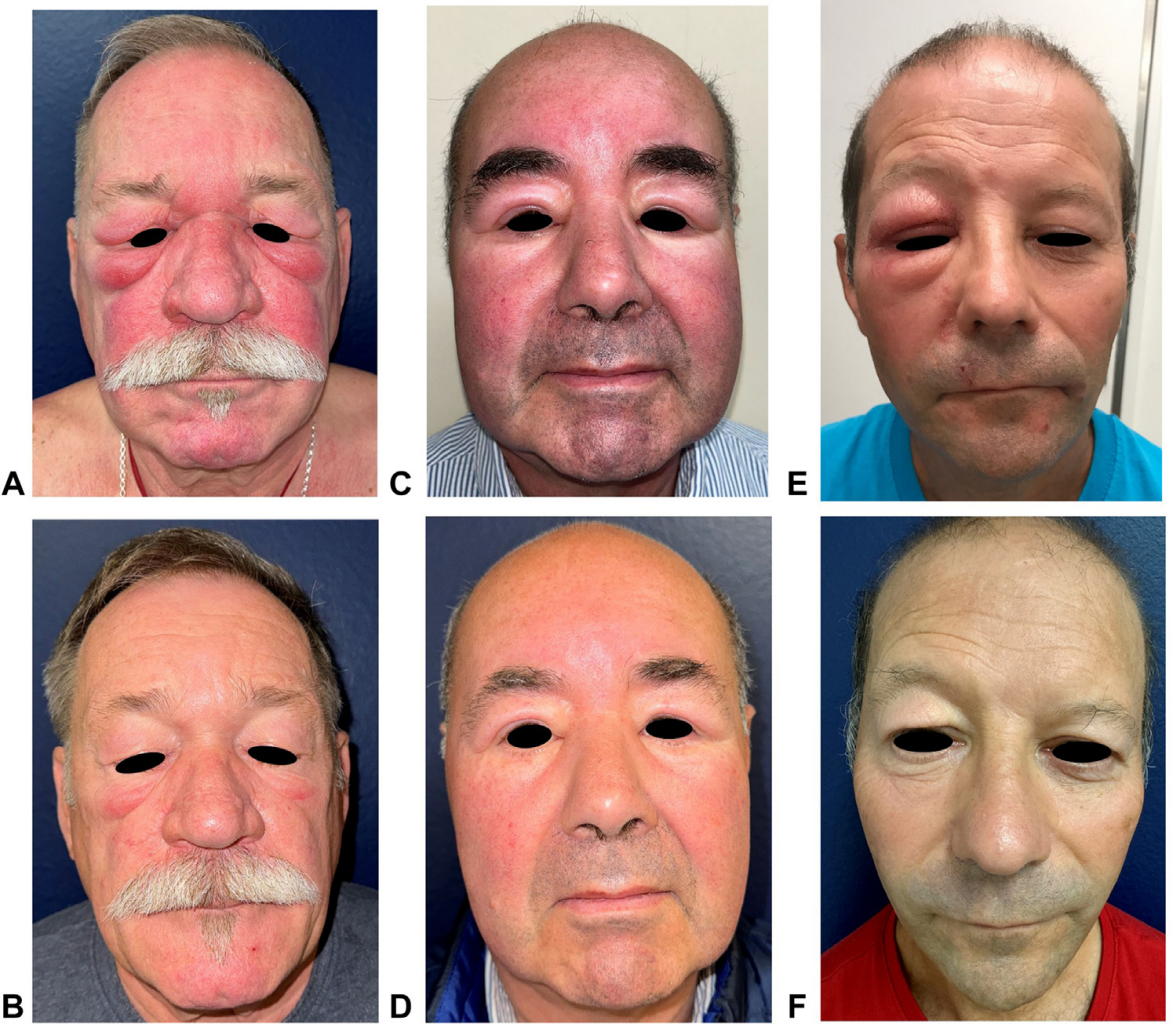
Clinical evolution of patients with solid facial edema (SFE) with abrocitinib 200 mg. Clinical aspect of patient 1 before **(A)** and after 9 months of treatment **(B)**, patient 2 before **(C)** and after 9 months of treatment **(D)**, and patient 3 before **(E)** and after 7.5 months of treatment **(F)**.

A 67-year-old White man presented with a 7-month history of progressive swelling and erythema of the centrofacial region, accompanied by severe edema of all 4 eyelids and intense pruritus (Fig 1, *A*). His medical history included hypertension, mild hypercholesterolemia, and ascending aortic aneurysm. Laboratory tests were unremarkable, and a skin biopsy showed vascular dilatation and mild dermal inflammatory infiltrate, mainly lymphohistiocytic, located around the hair follicles. Previous treatments, including systemic corticosteroids, isotretinoin, lymecycline, diuretics, oral metronidazole, topical ivermectin, and lymphatic drainage, had been unsuccessful. Treatment with abrocitinib 200 mg daily led to significant improvement in redness, edema, and pruritus (Fig 1, *B*), with only mild persistent erythroderma of the inferior eyelids 9 months after treatment initiation, and no adverse events.

### Case 2

Another 67-year-old White man presented with a 3-year history of worsening facial swelling and erythema (Fig 1, *C*). His medical history included asthma, hypertension, type II diabetes, and Parkinson’s disease. Laboratory tests were normal, and a skin biopsy showed a mild superficial intradermal perivascular lymphocytic infiltrate, associated with mild intradermal edema and focal parakeratosis. Previous treatments with systemic corticosteroids, isotretinoin, and doxycycline, had yielded poor results. Treatment with abrocitinib 200 mg daily for 9 months resulted in a significant reduction in edema and redness (Fig 1, *D*), with only a transient grade I thrombopenia observed.

### Case 3

An otherwise healthy 60-year-old White man presented with a 10-year history of persistent solid edema of the right eyelids, unresponsive to tetracycline and azathioprine (Fig 1, *E*). Magnetic resonance imaging of the orbital region and laboratory tests were normal. Skin biopsy showed edema with a moderate lymphohistiocytic perivascular and periadnexal dermal inflammatory infiltrate with nonnecrotic granulomas. Treatment with abrocitinib 200 mg daily led to a near-complete resolution of SFE after 7.5 months, with only a residual postedema blepharoptosis (Fig 1, *F*). No adverse events were reported. Discontinuation of treatment led to the relapse of SFE.

## Discussion

Although the histopathological features of SFE are not specific, the identification of granulomas as a key factor in SFE has expanded the therapeutic possibilities with the use of JAK inhibitors.^6^, ^7^, ^8^ In this observation, all 3 patients responded well to the treatment, even though granulomas were only observed in 1 patient, suggesting that their absence should not preclude the use of JAK inhibitors. The absence of granulomas could be explained by a biopsy being performed too early, insufficient depth, or ongoing anti-inflammatory medication.^1^ Moreover, the JAK/signal transducer and activator of transcription pathway play a pivotal role in rosacea^9^ where JAK 1 inhibitors have demonstrated efficacy by reducing inflammation, modulating vascular permeability, and inhibiting angiogenesis.^10^ Considering that SFE may be regarded as a complication of rosacea and that these same mechanisms are central to the pathophysiology of SFE, the observed effectiveness of abrocitinib in treating SFE, as herein reported, appears consistent.

Physicians and patients should also expect symptoms to improve more slowly than in atopic dermatitis or granulomatous cheilitis^5^ and be aware that slight residual edema or eyelid ptosis is possible. This minimal persistent symptomatology is counter-balanced by the significant improvement in patients’ quality of life.

These cases suggest that abrocitinib may be an effective treatment for SFE, providing significant improvement in symptoms, with a favorable safety profile. Longer-term and larger studies, also with other JAK inhibitors, are needed to further corroborate these findings.

## Conflicts of interest

Dr Baeck discloses her past participation on the abrocitinib advisory board organized by Pfizer. Dr De Greef has no conflicts of interest to declare.
